# Adaptive Regulation of mTOR Activity by AMPK, Akt, and ATF6 Pathways in Pi*Z Alpha-1 Antitrypsin Deficient Hepatocytes

**DOI:** 10.3390/biom16040506

**Published:** 2026-03-27

**Authors:** Yuanqing Lu, Jungnam Lee, Naweed Mohammad, Mark L. Brantly

**Affiliations:** Division of Pulmonary, Critical Care and Sleep Medicine, University of Florida College of Medicine, 1600 SW Archer Rd., Room M330, J. Hillis Miller Health Center (JHMHC), Gainesville, FL 32610, USA

**Keywords:** alpha-1 antitrypsin deficiency, mTOR, AMPK, Akt, ATF6, ER stress, autophagy, hepatocytes

## Abstract

Alpha-1 antitrypsin deficiency (AATD) is an inherited disorder characterized by intracellular retention of mutant Z (Pi*Z) alpha-1 antitrypsin (AAT) within hepatocytes, resulting in progressive liver disease. Currently, no approved pharmacological therapies exist for AATD-associated hepatic injury. Emerging preclinical evidence indicates that inhibition of mammalian target of rapamycin (mTOR) ameliorates liver pathology in AATD; however, the status of mTOR activity and its regulatory mechanisms under Pi*Z AAT-induced cellular stress remains incompletely understood. In this study, we investigated alterations in mTOR signaling and its upstream regulatory pathways using a gene-edited human hepatocyte model harboring the Pi*Z mutation (Huh7.5Z cells) and a Pi*Z AAT transgenic mouse model. Attenuation of mTORC1 activity was observed in both cellular and murine Pi*Z models. In vitro analyses demonstrated activation of AMP-activated protein kinase (AMPKα), a key inhibitory regulator of mTORC1, accompanied by paradoxical activation of Akt and the unfolded protein response (UPR) branch ATF6α. Pharmacological inhibition of mTOR significantly reduced intracellular Pi*Z AAT accumulation, alleviated ER stress, and suppressed apoptotic signaling through enhancement of autophagy. These findings reveal that hepatocytes adapt to Pi*Z AAT-induced stress through coordinated regulation of mTOR by AMPK, Akt, and ATF6α pathways. This study provides mechanistic insight into metabolic and stress-response signaling in AATD and identifies mTOR modulation as a promising therapeutic strategy for AATD-associated liver disease.

## 1. Introduction

Alpha-1 antitrypsin (AAT) is a highly abundant circulating glycoprotein belonging to the serine protease inhibitor family and is synthesized predominantly by hepatocytes. It is encoded by the *SERPINA1* gene. The Pi*Z (*Glu342Lys*) mutation represents the most common pathogenic variant associated with severe alpha-1 antitrypsin deficiency (AATD) [1]. This mutation promotes protein misfolding, polymerization, and aggregation within the endoplasmic reticulum (ER), leading to hepatocellular injury. Homozygous Pi*Z AATD is the most frequent genetic cause of pediatric liver disease [2,3], and approximately one-third of adults with Pi*Z AATD develop significant liver fibrosis [4,5]. Additional pathological features include hepatic steatosis, impaired lipid secretion, inflammation, and hepatocyte apoptosis [6,7].

The mammalian target of rapamycin (mTOR), comprising mTOR complex 1 (mTORC1) and mTOR complex 2 (mTORC2), functions as a central integrator of nutrient availability, cellular energy status, and hormonal signals to regulate growth, metabolism, autophagy, and survival [8,9]. mTOR signaling also plays a critical role in fibrogenesis and tissue remodeling [10]. Two principal upstream regulators of mTOR are AMP-activated protein kinase (AMPK) and protein kinase B (Akt) [11]. AMPK acts as a cellular energy sensor and suppresses mTOR activity during metabolic stress, whereas Akt activates mTOR in response to growth factor signaling.

The unfolded protein response (UPR) is an adaptive program that mitigates ER stress by restoring protein-folding homeostasis. Activation of UPR signaling has been demonstrated in Pi*Z AATD [12]. The UPR consists of three major branches initiated by ER-resident transmembrane sensors: protein kinase R-like ER kinase (PERK), inositol-requiring enzyme 1α (IRE1α), and activating transcription factor 6α (ATF6α) [13]. Increasing evidence suggests that ATF6α can directly regulate mTOR signaling, thereby linking ER stress responses to metabolic control pathways [14,15,16].

mTOR rapidly responds to ER stress [17] and mitochondrial dysfunction [18], both of which are hallmark pathological features of Pi*Z AATD [19]. Autophagy is the primary degradative pathway responsible for the clearance of aggregated Pi*Z AAT in hepatocytes [20,21]. Importantly, mTORC1 is a negative regulator of autophagy [22]. Pharmacological inhibition of mTOR reduces intracellular Pi*Z AAT accumulation in both cellular and animal models [23,24], highlighting a critical role for mTOR in disease pathogenesis and identifying it as a potential therapeutic target.

Despite these observations, the adaptive regulation of mTOR signaling under chronic Pi*Z AAT stress remains poorly defined. In particular, the coordinated roles of the AMPK, Akt, and UPR pathways in regulating mTOR activity in Pi*Z hepatocytes have not been systematically investigated.

In this study, we examined changes in mTORC1 activity and its regulatory networks using a gene-edited human hepatocyte model expressing the Pi*Z mutation and a Pi*Z AAT transgenic mouse model. We focused on the interplay between metabolic stress signaling (AMPK and Akt) and ER stress pathways (UPR). Our findings demonstrate that mTORC1 activity is suppressed in Pi*Z hepatocytes and mouse liver tissue through coordinated regulation by AMPK, Akt, and ATF6α signaling, thereby enhancing autophagy and limiting intracellular AAT accumulation. These results reveal a complex adaptive network that balances metabolism, stress responses, and cell survival in AATD.

## 2. Materials and Methods

### 2.1. Cell Culture

Huh7.5Z cells, a human hepatoma cell line carrying the Pi*Z mutation in the *SERPINA1* gene, were generated from parental Huh7.5 cells (passage 16) using CRISPR/Cas9 gene editing as previously described [9]. Huh7.5 and Huh7.5Z cells were cultured in high-glucose DMEM (4.5 g/L; Thermo Fisher Scientific, Waltham, MA, USA) supplemented with 10% fetal bovine serum (FBS) at 37 °C in a humidified incubator with 5% CO_2_. In this study, Huh7.5Z and Huh7.5 cells were generally used at passages below 30.

#### 2.1.1. Pharmacological Inhibitors

- Akt inhibition: AKTi-1/2 (10 μM, 18 h; MedChemEXpress, Monmouth Junction, NJ, USA).
- AMPK inhibition: dorsomorphin (10 μM, 24 h; MedChemEXpress, Monmouth Junction, NJ, USA).
- UPR inhibition: 4-phenylbutyric acid (1–10 mM, 12 h; MilliporeSigma, Burlington, MA, USA).
- IRE1α inhibition: KIRA6 (0.1–1 μM, 16 h; MilliporeSigma, Burlington, MA, USA).
- ATF6α inhibition: Ceapin-A7 (10 μM, 24 h; MedChemEXpress, Monmouth Junction, NJ, USA).
- mTOR inhibition: rapamycin (50 nM, 16 h; MedChemEXpress, Monmouth Junction, NJ, USA).

Equivalent volumes of DMSO served as vehicle controls.

#### 2.1.2. siRNA Transfection

PERK knockdown was performed using SignalSilence^®^ PERK siRNA (50 nM; Cell Signaling Technology, Danvers, MA, USA). A validated control siRNA was used as a negative control. Transfections were conducted using GenMute™ transfection reagent according to the manufacturer’s protocol. Cells were harvested 48 h post-transfection.

### 2.2. Transgenic Mouse Models

Pi*Z (C57BL/6J-Tg (SERPINA1*E366K) 1Mib/J, Jackson lab stock No. 037670) and PiM (C57BL/6J-Tg (*SERPINA1*)1Mlb/Jackson lab stock No. 037669) transgenic mice were maintained under specific pathogen-free conditions at 22  ±  3 °C with a 12:12-h light–dark cycle. Mice were housed in ventilated cages with an automatic watering system and with a standard diet, corn cob bedding, and standard enrichment. Six- to twelve-month-old mice were euthanized, and liver tissues were harvested and stored at −80 °C. Animal protocols were approved by the University of Florida IACUC. Only male mice were used in this study, as significant sex-dependent differences in hAAT expression and liver pathological phenotypes have been reported in hAAT transgenic mouse models [25].

### 2.3. Western Blot Analysis

Cellular and liver tissue lysates were prepared using radioimmunoprecipitation assay (RIPA) buffer supplemented with protease/phosphatase inhibitor cocktail and Ambion DNase I. Samples were homogenized using a 2010 Geno/Grinder homogenizer (Spex SamplePrep, Metuchen, NJ, USA) and subsequently sonicated in a bath sonicator for 2 min (cell lysates) or 5 min (tissue lysates) to ensure complete lysis and DNA fragmentation.

Lysates were centrifuged to remove insoluble debris, and the supernatants were collected for protein analysis. Protein samples were mixed 1:1 with 2 × Laemmli sample loading buffer containing 5% 2-mercaptoethanol and heat-denatured at 95 °C for 10 min.

Equal amounts of protein were separated by SDS–PAGE using Criterion™ Tris-glycine extended (TGX) 4–15% precast midi gels. Proteins were then transferred onto nitrocellulose membranes (0.45 µm or 0.2 µm for low molecular weight proteins). After transfer, membranes were cut horizontally according to the expected molecular weights of the target proteins, allowing the simultaneous detection of multiple proteins from the same sample.

Membranes were blocked with 5% blocking buffer (non-fat milk in TBST) or 5% FBS in TBST for the detection of phosphorylated proteins. Membranes were incubated overnight at 4 °C with the appropriate primary antibodies (Table S1). Following primary antibody incubation, membranes were washed with TBST and incubated with horseradish peroxidase (HRP)-conjugated secondary antibodies (Table S1).

Protein bands were detected using chemiluminescent substrates, either Clarity™ Max Western ECL substrate or Lumigen ECL Ultra for weak signals. Chemiluminescent signals were captured using a ChemiDoc Touch Imaging System (Bio-Rad, Hercules, CA, USA).

Protein band intensities were quantified by densitometry using ImageJ 15.4g. The levels of target proteins were normalized to the corresponding loading controls.

Some blots were re-probed after stripping with Thermo Scientific™ Restore™ Western Blot Stripping Buffer according to the manufacturer’s instructions.

### 2.4. RT-qPCR

RNA was isolated using the RNeasy Plus Micro Kit and reverse-transcribed to cDNA. Quantitative PCR was performed using TaqMan probes (Table S2). Gene expression was normalized to 18S rRNA and calculated using the 2^−ΔΔCt^ method.

### 2.5. Immunofluorescence

Cells were fixed with 4% formaldehyde, permeabilized with Triton X-100, and incubated with primary and fluorescent secondary antibodies. Nuclei were counterstained with DAPI. Images were acquired using a Keyence fluorescence microscope (Keyence, Itasca, IL, USA).

### 2.6. ADP/ATP Ratio Assay

Cellular ADP/ATP ratios were measured using a bioluminescence assay kit according to the manufacturer’s instructions.

### 2.7. Statistical Analysis

Data are presented as mean ± SD. Comparisons between two groups were performed using Student’s *t*-test. Multiple group comparisons were analyzed by one-way ANOVA with Dunnett’s post hoc test. Statistical significance was defined as *p* < 0.05.

## 3. Results

### 3.1. mTORC1 Activity Is Suppressed Through AMPK Signaling in Huh7.5Z Cells and Pi*Z Transgenic Mouse Liver

**Figure 1:**
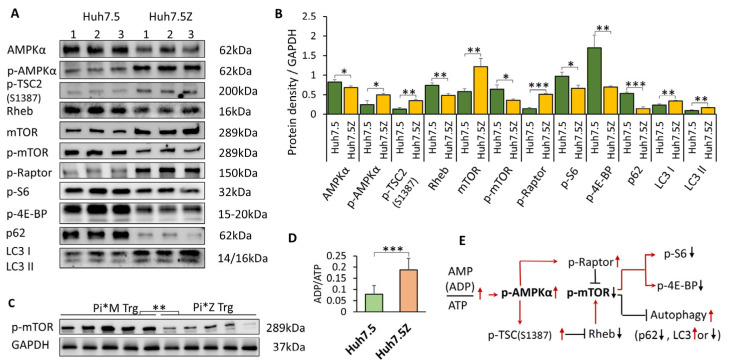
Suppression of mTOR activity and activation of AMPK signaling in Huh7.5Z cells. Huh7.5Z and Huh7.5 cells were cultured in high-glucose complete DMEM, and cell lysates were subjected to Western blot analysis. (**A**) Representative Western blots of AMPKα, p-AMPKα, p-TSC2 (Ser1387), Rheb, mTOR, p-mTOR, p-Raptor, p-S6, p-4E-BP1, p62, LC3-I, and LC3-II in Huh7.5 and Huh7.5Z cells. Images were obtained from four independent blots. Original Western blot images, including loading controls, are provided in Supplementary Figure S1A–D. (**B**) Quantification of protein expression normalized to GAPDH on the same blot (*n* = 3 independent experiments). (**C**) Western blot analysis of p-mTOR in liver lysates from five male Pi*Z hAAT transgenic mice and five male Pi*M hAAT transgenic mice used as controls (6–12 months old). Quantification was normalized to GAPDH. Original Western blot images are provided in Supplementary Figure S1E. (**D**) ADP/ATP ratio in Huh7.5 and Huh7.5Z cells (*n* = 10 independent experiments). (**E**) Schematic representation of AMPK–mTOR signaling. Arrows indicate activation, and T-shaped lines indicate inhibition. Red and black denote upregulation and downregulation, respectively. Data are presented as mean ± SD. Statistical significance was determined using Student’s *t*-test (* *p* < 0.05, ** *p* < 0.01, *** *p* < 0.001).

Phosphorylated mTOR (p-mTOR) represents the activated form of mTOR and serves as an indicator of mTORC1 activity. Western blot analysis demonstrated a significant reduction in p-mTOR levels in Huh7.5Z cells compared with parental Huh7.5 cells (*p* < 0.05; Figure 1A,B). Consistently, liver lysates from Pi*Z human AAT transgenic mice exhibited significantly lower p-mTOR levels compared with Pi*M control mice (*p* < 0.01; Figure 1C).

To further confirm suppression of mTORC1 signaling, we measured two downstream targets of mTORC1: phosphorylated ribosomal protein S6 (p-S6) and phosphorylated eukaryotic translation initiation factor 4E-binding protein (p-4E-BP). Both p-S6 and p-4E-BP were significantly reduced in Huh7.5Z cells relative to controls (*p* < 0.05 and *p* < 0.01, respectively; Figure 1A,B).

Because mTORC1 negatively regulates autophagy, we assessed autophagic activity by measuring LC3 and p62 protein levels. Huh7.5Z cells displayed increased levels of LC3-I and LC3-II (*p* < 0.01 for both) and a marked reduction in the autophagy substrate p62 (*p* < 0.001; Figure 1A,B), indicating enhanced autophagic flux.

Measurement of cellular energy status revealed a significantly increased ADP/ATP ratio in Huh7.5Z cells compared with Huh7.5 cells (*p* < 0.001; Figure 1D), consistent with mitochondrial dysfunction. Correspondingly, phosphorylated AMPKα (p-AMPKα) was significantly increased in Huh7.5Z cells (*p* < 0.05).

AMPK inhibits mTORC1 through directly phosphorylating the Raptor subunit of mTORC1 [26,27] and phosphorylating the tuberous sclerosis complex (TSC)2 tumor suppressor (at Ser1387) [28] to inhibit Rheb activity (Figure 1E). Western blot analysis demonstrated increased phosphorylation of TSC2 at Ser1387 (*p* < 0.01), reduced Rheb levels (*p* < 0.01), and increased phosphorylated Raptor (*p* < 0.001) in Huh7.5Z cells compared with controls (Figure 1A,B). These findings indicate that mitochondrial dysfunction activates the AMPK pathway, leading to suppression of mTORC1 activity in Pi*Z hepatocytes.

Interestingly, total mTOR protein levels were increased (*p* < 0.01), whereas total AMPKα levels were decreased (*p* < 0.05) in Huh7.5Z cells, suggesting the presence of compensatory feedback regulation within the AMPK–mTOR signaling network [29].

### 3.2. Akt/mTOR Signaling Is Paradoxically Activated in Huh7.5Z Cells

**Figure 2:**
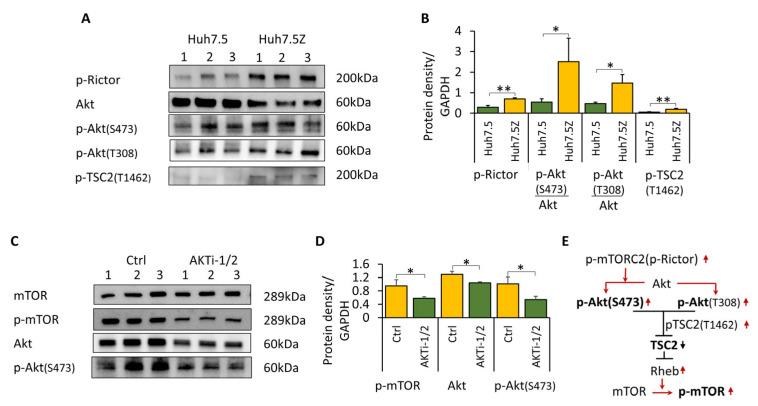
Activation of Akt signaling despite suppression of mTORC1 in Huh7.5Z cells. Huh7.5Z and Huh7.5 cells were cultured in high-glucose complete DMEM, and cell lysates were subjected to Western blot analysis. (**A**) Representative Western blots of p-Rictor, Akt, p-Akt (Ser473), p-Akt (Thr308), and p-TSC2 (Thr1462). Images were obtained from three independent blots. Original Western blot images, including loading controls, are provided in Supplementary Figure S2A–C. (**B**) Quantification of protein expression normalized to GAPDH on the same blot (*n* = 3 independent experiments). (**C**,**D**) Effects of Akt inhibition (AKTi-1/2) on mTOR and Akt signaling. Huh7.5Z cells were treated with 10 μM AKTi-1/2 for 18 h, and cell lysates were subjected to Western blot analysis. (**C**) Representative Western blots of mTOR, p-mTOR, Akt, and p-Akt (Ser473). Images were obtained from two independent blots. Original images, including loading controls, are provided in Supplementary Figure S2D,E. (**D**) Quantification of protein expression normalized to GAPDH on the same blot (*n* = 3 independent experiments). (**E**) Schematic diagram of Akt–mTOR signaling interactions. Arrows indicate activation, and T-shaped lines indicate inhibition. Red and black denote upregulation and downregulation, respectively. Data are presented as mean ± SD. Statistical significance was determined using Student’s *t*-test (* *p* < 0.05, ** *p* < 0.01).

TSC2 phosphorylation at Thr1462 inhibits TSC2 activity and promotes mTORC1 activation. Despite increased phosphorylation of TSC2 at Ser1387, phosphorylation at Thr1462 was also significantly elevated in Huh7.5Z cells (*p* < 0.01; Figure 2A,B), indicating bidirectional regulation of TSC2.

Because TSC2 Thr1462 is primarily phosphorylated by Akt, we next examined Akt activation. Although total Akt protein levels were reduced in Huh7.5Z cells, relative phosphorylation of Akt at Ser473 and Thr308 was significantly increased (*p* < 0.05 for both; Figure 2A,B), demonstrating enhanced Akt activation.

Akt Ser473 phosphorylation is mediated by mTORC2 via its core subunit Rictor [30]. Consistent with this mechanism, phosphorylated Rictor (p-Rictor) levels were significantly elevated in Huh7.5Z cells (*p* < 0.01; Figure 2A,B). This increase in mTORC2–Akt signaling is consistent with suppression of mTORC1, as mTORC1 negatively regulates mTORC2 through feedback inhibition [31].

To directly assess the contribution of Akt to mTOR regulation, Huh7.5Z cells were treated with the Akt inhibitor AKTi-1/2. Inhibition of Akt significantly reduced p-mTOR levels (*p* < 0.05; Figure 2C,D), confirming that Akt signaling contributes positively to mTORC1 activity in Pi*Z hepatocytes.

These findings suggest that although AMPK-mediated inhibition dominates overall mTORC1 suppression, Akt signaling remains activated and partially counteracts mTORC1 inhibition in Huh7.5Z cells.

### 3.3. Inhibition of AMPK Further Suppresses mTOR Activity via Downregulation of Akt Signaling

**Figure 3:**
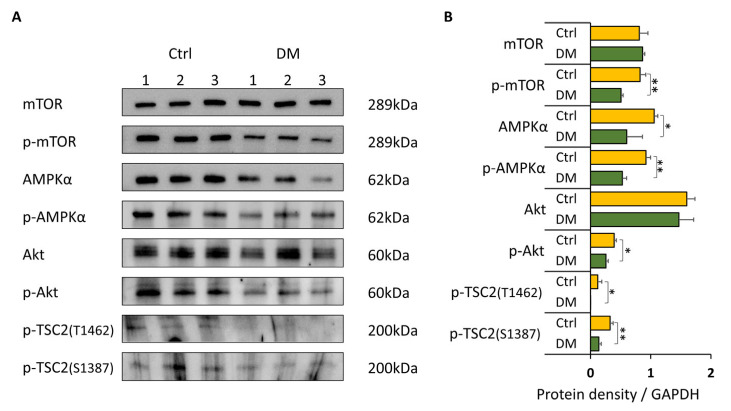
Inhibition of AMPK suppresses Akt activation and further reduces mTOR activity. Huh7.5Z cells were treated with 10 μM dorsomorphin (DM) for 18 h in complete DMEM, and cell lysates were subjected to Western blot analysis. (**A**) Representative Western blots of mTOR, p-mTOR, AMPKα, p-AMPKα, Akt, p-Akt, p-TSC2 (Thr1462), and p-TSC2 (Ser1387) following dorsomorphin treatment. Images were obtained from two independent blots. Original images, including loading controls, are provided in Supplementary Figure S3A,B. (**B**) Quantification of protein expression normalized to GAPDH on the same blot (*n* = 3 independent experiments). Data are presented as mean ± SD. Statistical significance was determined using Student’s *t*-test (* *p* < 0.05, ** *p* < 0.01).

To determine the effect of AMPK inhibition on mTOR signaling, Huh7.5Z cells were transfected with 50 nM AMPKα2 siRNA (SignalSilence^®^ AMPKα2 siRNA II) for 24 h, with 50 nM scrambled siRNA used as a negative control. Unexpectedly, silencing of AMPK using siRNA in Huh7.5Z cells reduced p-mTOR levels (Figure S8). Because AMPK siRNA induced significant cell death in Huh7.5Z cells, we next used the AMPK inhibitor dorsomorphin (DM) to further investigate this effect. DM treatment significantly reduced p-AMPKα levels (*p* < 0.05) and led to decreased phosphorylation of TSC2 at Ser1387 (*p* < 0.01; Figure 3A,B). Consistent with the AMPK siRNA results, p-mTOR levels were further reduced following DM treatment (*p* < 0.01; Figure 3A,B). This paradoxical result was accompanied by significant reductions in p-Akt and p-TSC2 (Thr1462) (*p* < 0.05; Figure 3A,B).

Although dorsomorphin does not directly inhibit Akt, previous studies have shown that Akt activation can be regulated through the AMPK-dependent PI3K/Akt and mTORC2/Akt pathways [25,26]. Thus, inhibition of AMPK in Huh7.5Z cells indirectly suppressed Akt signaling, resulting in further reduction in mTORC1 activity.

These findings demonstrate that coordinated regulation of the AMPK and Akt pathways is required to maintain adaptive mTOR signaling under Pi*Z AAT-induced stress.

### 3.4. UPR Signaling Contributes to Regulation of mTORC1 in Huh7.5Z Cells

**Figure 4:**
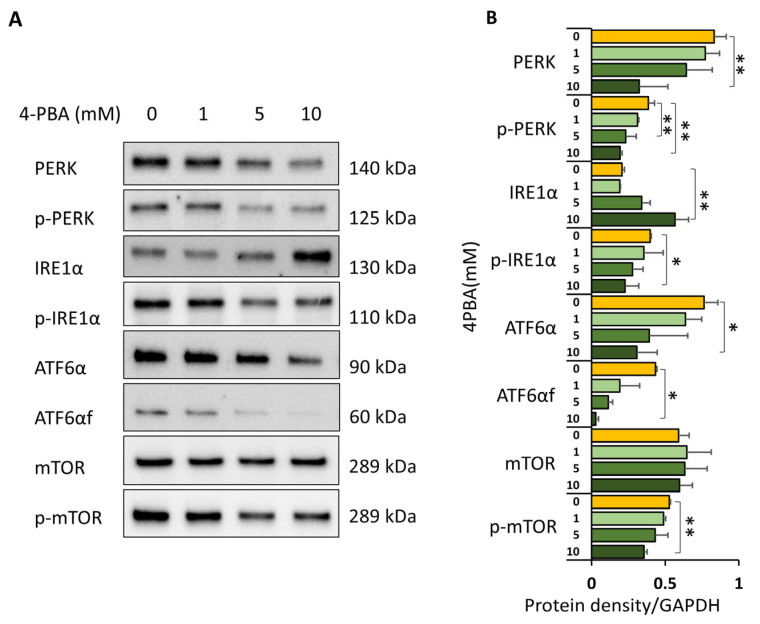
Broad inhibition of the UPR reduces mTOR activity in Huh7.5Z cells. Huh7.5Z cells were treated with 10 mM 4-phenylbutyric acid (4-PBA) for 12 h in complete DMEM, and cell lysates were subjected to Western blot analysis. (**A**) Representative Western blots of PERK, p-PERK, IRE1α, p-IRE1α, ATF6α, cleaved ATF6α (ATF6αf), mTOR, and p-mTOR following 4-PBA treatment. Images were obtained from four independent blots. Original images, including loading controls, are provided in Supplementary Figure S4A–D. (**B**) Quantification of protein expression normalized to GAPDH on the same blot (*n* = 3 independent experiments). Data are presented as mean ± SD. Statistical significance was determined using one-way ANOVA (* *p* < 0.05, ** *p* < 0.01).

To assess the role of UPR signaling in mTOR regulation, Huh7.5Z cells were treated with 4-phenylbutyric acid (4-PBA), a chemical chaperone that broadly suppresses all three branches of the UPR. Western blot analysis confirmed dose-dependent reductions in phosphorylated PERK, phosphorylated IRE1α, and cleaved ATF6α following 4-PBA treatment (Figure 4A,B). Notably, inhibition of UPR signaling resulted in a significant reduction in p-mTOR levels (*p* < 0.01; Figure 4A,B), indicating that UPR activation contributes positively to mTORC1 activity in Pi*Z hepatocytes.

**Figure 5:**
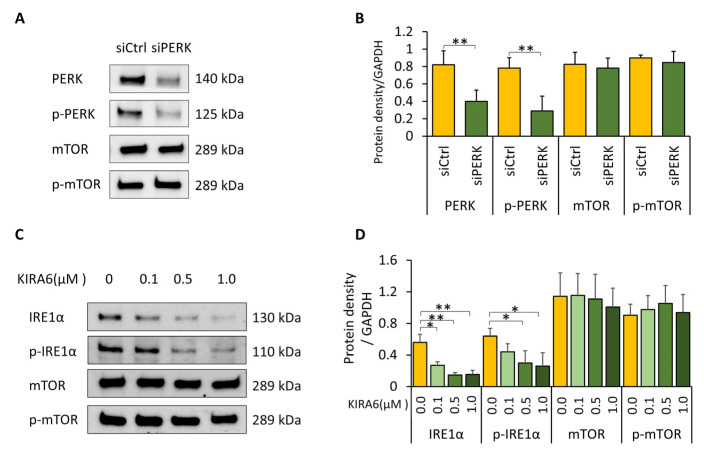
PERK and IRE1α branches do not regulate mTOR activity in Pi*Z hepatocytes. (**A**,**B**) Huh7.5Z cells were transfected with 50 nM PERK siRNA or control siRNA for 48 h, and cell lysates were subjected to Western blot analysis. (**A**) Representative Western blots of PERK, p-PERK, mTOR, and p-mTOR. Images were obtained from two independent blots. Original images, including loading controls, are provided in Supplementary Figure S5A,B. (**B**) Quantification of protein expression normalized to GAPDH on the same blot (*n* = 3 independent experiments). (**C**,**D**) Effects of IRE1α inhibition by KIRA6 on mTOR activity. Huh7.5Z cells were treated with 0.1, 0.5, or 1.0 μM KIRA6, with the same amount of DMSO used as the vehicle control, for 16 h. Cell lysates were subjected to Western blot analysis. (**C**) Representative Western blots of IRE1α, p-IRE1α, mTOR, and p-mTOR. Images were obtained from two independent blots. Original images, including loading controls, are provided in Supplementary Figure S5C,D. (**D**) Quantification of protein expression normalized to GAPDH on the same blot (*n* = 3 independent experiments). Data are presented as mean ± SD. Statistical significance was determined using Student’s *t*-test (**A**) and one-way ANOVA (**B**) (* *p* < 0.05, ** *p* < 0.01).

To determine which UPR branch mediated this effect, individual pathways were selectively inhibited. Knockdown of PERK using siRNA effectively reduced PERK and p-PERK levels but did not alter p-mTOR expression (Figure 5A,B). Similarly, pharmacological inhibition of IRE1α using KIRA6 significantly reduced IRE1α and p-IRE1α levels without affecting mTOR activity (Figure 5C,D). Of note, we observed a parallel reduction in total IRE1α following KIRA6 treatment, like the decrease in total AMPKα observed after dorsomorphin (DM) treatment. The mechanism underlying this reduction in total protein levels is not fully understood. It may reflect reduced protein stability and enhanced degradation following kinase binding with small-molecule inhibitors.

**Figure 6:**
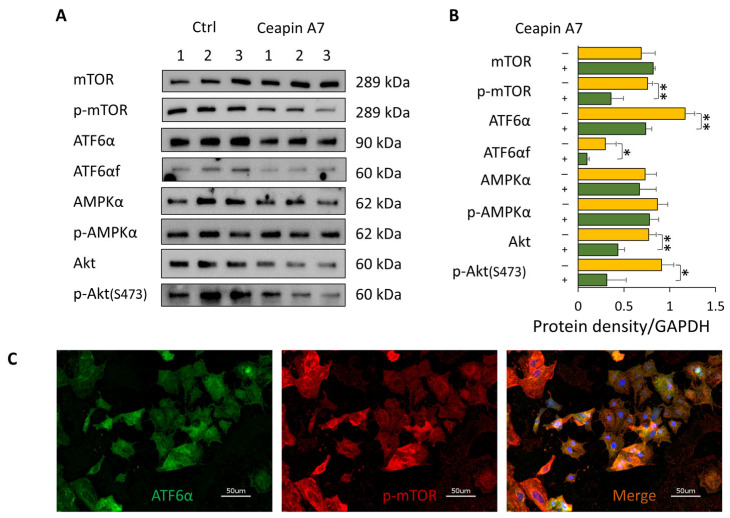
ATF6α regulates mTOR activity through Akt signaling. Huh7.5Z cells were treated with 10 μM Ceapin-A7 for 24 h in complete medium, and cell lysates were subjected to Western blot analysis. (**A**) Representative Western blots of mTOR, p-mTOR, ATF6α, cleaved ATF6α (ATF6αf), AMPK, p-AMPK, Akt, and p-Akt (Ser473). Images were obtained from five independent blots. Original images, including loading controls, are provided in Supplementary Figure S6A–E. (**B**) Quantification of protein expression normalized to GAPDH on the same blot (*n* = 3 independent experiments). Data are presented as mean ± SD. Statistical significance was determined using Student’s *t*-test (* *p* < 0.05, ** *p* < 0.01). (**C**) Immunofluorescence staining of Huh7.5Z cells showing a cell-level correlation between ATF6α (green) and p-mTOR (red). Nuclei were counterstained with DAPI (blue). Scale bar: 50 μm. Magnification: 40×.

In contrast, inhibition of ATF6α using Ceapin-A7 significantly reduced ATF6α and its cleaved active fragment, ATF6αf, and was accompanied by a marked decrease in p-mTOR levels (*p* < 0.01; Figure 6A,B). These results identify ATF6α as the primary UPR branch regulating mTORC1 activity in Huh7.5Z cells.

Further analysis revealed that AMPKα and p-AMPKα levels were unchanged following ATF6α inhibition, whereas Akt and p-Akt levels were significantly reduced (*p* < 0.01 and *p* < 0.05, respectively; Figure 6A,B), indicating that ATF6α regulates mTORC1 through an Akt-dependent mechanism.

Immunofluorescence showed that cells with higher ATF6α levels also exhibited higher p-mTOR levels, supporting a functional link between ATF6α signaling and mTOR activation (Figure 6C).

### 3.5. mTOR Inhibition Reduces Pi*Z AAT Accumulation, ER Stress, and Apoptosis in Huh7.5Z Cells

**Figure 7:**
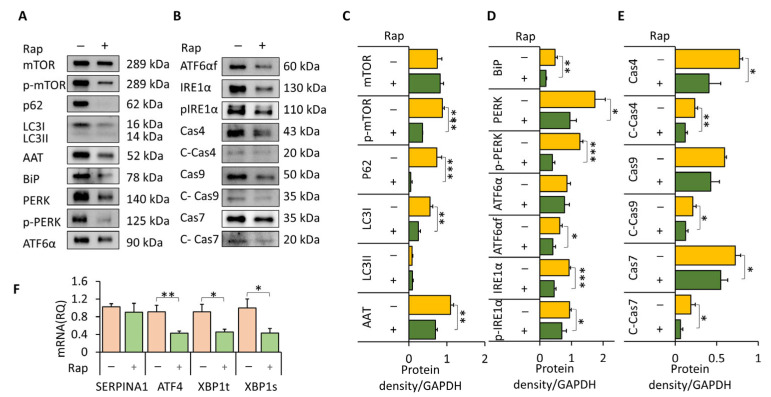
mTOR inhibition enhances autophagy and reduces intracellular Pi*Z AAT accumulation, ER stress, and apoptosis. Huh7.5Z cells were treated with 50 nM rapamycin or an equivalent amount of DMSO as the vehicle control in complete DMEM for 16 h. Cell lysates were subjected to Western blot analysis, and total RNA was used for RT-qPCR analysis. (**A**,**B**) Representative Western blots of intracellular AAT and mTORC1-, UPR-, autophagy-, and apoptosis-related proteins following rapamycin treatment. Images were obtained from four independent blots. Original images, including loading controls, are provided in Supplementary Figure S7A–D. Some proteins were re-probed after membrane stripping, as shown in Supplementary Figure S7E. (**C**–**E**) Quantification of protein expression normalized to GAPDH on the same blot (*n* = 3 independent experiments). (**F**) RT-qPCR analysis of *SERPINA1, ATF4, XBP1t,* and *XBP1s* mRNA expression levels. Data are presented as mean ± SD. Statistical significance was determined using Student’s *t*-test (* *p* < 0.05, ** *p* < 0.01, *** *p* < 0.001).

To investigate the functional consequences of mTOR inhibition, Huh7.5Z cells were treated with rapamycin. Rapamycin significantly reduced p-mTOR levels (*p* < 0.001; Figure 7A,C) and enhanced autophagic activity, as evidenced by decreased p62 and LC3 protein levels (*p* < 0.001 and *p* < 0.01, respectively; Figure 7A,C).

Rapamycin treatment significantly reduced intracellular AAT protein levels (*p* < 0.01; Figure 7A,C), whereas *SERPINA1* mRNA expression remained unchanged (Figure 7F), indicating that AAT reduction occurred primarily through enhanced protein degradation rather than transcriptional regulation.

**Figure 8:**
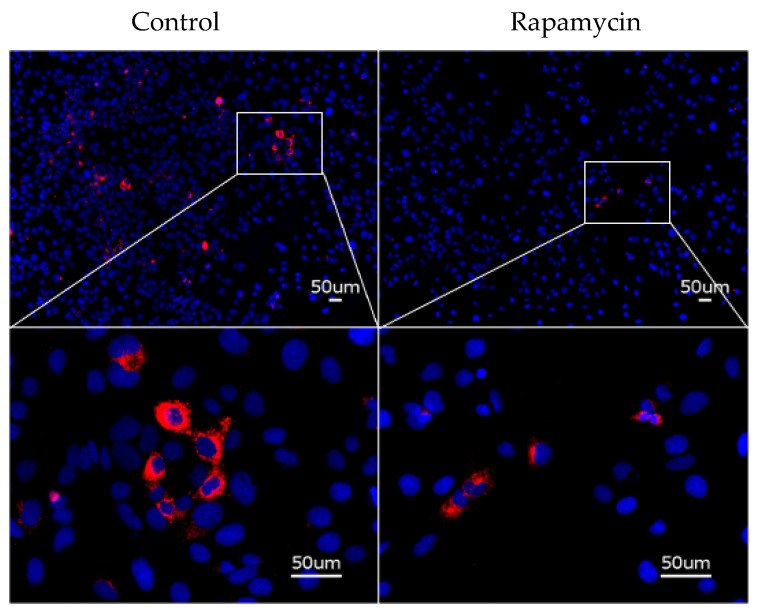
Immunofluorescence staining of intracellular AAT polymers using the 2C1 antibody. Huh7.5Z cells were treated with 50 nM rapamycin or an equivalent volume of DMSO, which served as vehicle controls for 16 h. Immunofluorescence staining using the 2C1 antibody showed that AAT polymer-positive cells accounted for 5.3% of rapamycin-treated Huh7.5Z cells, compared with 23.8% in untreated Huh7.5Z cells (*p* < 0.01). Scale bar: 50 μm. Magnification: **upper panels**, 20×; **lower panels**, 40×.

Immunofluorescence staining using the AAT polymer-specific antibody 2C1 demonstrated a marked reduction in polymer-positive cells from 23.8% in untreated Huh7.5Z cells to 5.3% following rapamycin treatment (*p* < 0.01; Figure 8).

mTOR inhibition also significantly attenuated ER stress signaling. Protein levels of BiP, PERK, phosphorylated PERK, cleaved ATF6α, IRE1α, and phosphorylated IRE1α were significantly reduced following rapamycin treatment (Figure 7A,B,D). Consistently, mRNA levels of *ATF4*, total *XBP1* (*XBP1t*), and spliced *XBP1* (*XBP1s*) were significantly decreased (Figure 7F).

Finally, rapamycin significantly suppressed apoptotic signaling, as demonstrated by reduced levels of caspase-4, cleaved caspase-4, cleaved caspase-9, caspase-7, and cleaved caspase-7 (Figure 7B,E).

Collectively, these results indicate that mTOR inhibition enhances autophagy, reduces intracellular Pi*Z AAT polymer accumulation, alleviates ER stress, and suppresses apoptosis in Pi*Z hepatocytes.

## 4. Discussion

Previous studies have demonstrated that pharmacological inhibition of mTOR reduces intracellular accumulation of Pi*Z AAT polymers and ameliorates liver injury in experimental models of AATD [23,24]. These findings underscore the importance of mTOR signaling in the pathogenesis of Pi*Z AAT–associated liver disease. However, the adaptive changes in mTOR activity and the upstream regulatory mechanisms triggered by chronic Pi*Z AAT accumulation in hepatocytes have remained largely undefined. In the present study, we provide a comprehensive analysis of mTOR regulation in Pi*Z hepatocellular and murine models and demonstrate that mTORC1 activity is suppressed through coordinated regulation by AMPK and ATF6α via Akt pathways.

TSC2 is a central regulator of mTORC1 activity [32]. Phosphorylation of TSC2 at distinct residues produces opposing effects on mTOR signaling. Phosphorylation at Ser1387 by AMPK inhibits mTORC1, whereas phosphorylation at Thr1462 by Akt suppresses TSC2 function and promotes mTORC1 activation [33]. Our data reveal a paradoxical increase in phosphorylation at both Ser1387 and Thr1462 in Huh7.5Z cells, indicating bidirectional regulation of TSC2. This dual regulation reflects the competing influences of metabolic stress and survival signaling in Pi*Z hepatocytes.

AMPK activation observed in this study is consistent with increased ADP/ATP ratios and mitochondrial dysfunction in Pi*Z hepatocytes. Mitochondrial injury has been recognized as a key pathological feature of AATD-associated liver disease [19,34]. Activation of AMPK under these conditions likely represents an adaptive response that suppresses mTORC1 to conserve energy and promote autophagy, thereby facilitating degradation of aggregated Pi*Z AAT. This mechanism supports cell survival under chronic proteotoxic stress.

In contrast, Akt signaling was also activated in Huh7.5Z cells despite reduced total Akt protein levels. Increased phosphorylation of Akt at Ser473 and Thr308, together with enhanced activation of Rictor (mTORC2), suggests that mTORC2-Akt signaling is upregulated when mTORC1 activity is suppressed. This observation is consistent with established negative feedback regulation between mTORC1 and mTORC2 [35,36]. Akt activation likely serves as a compensatory pro-survival mechanism that partially counterbalances AMPK-mediated inhibition of mTORC1.

Our findings further demonstrate that inhibition of AMPK paradoxically resulted in further suppression of mTOR activity. This effect was associated with decreased Akt phosphorylation, indicating that AMPK contributes indirectly to Akt activation through the AMPK–PI3K–Akt and AMPK–mTORC2–Akt pathways [37,38,39]. These results highlight the complexity of the regulatory network governing mTOR signaling and emphasize that AMPK and Akt pathways do not function independently but rather form an interconnected signaling circuit that fine-tunes mTOR activity in response to cellular stress.

Endoplasmic reticulum stress and activation of the UPR are central pathological processes in Pi*Z AATD [12]. Although previous studies have implicated PERK and IRE1α in mTOR regulation [40,41], our data indicate that neither the PERK nor the IRE1α branch significantly influences mTORC1 activity in Pi*Z hepatocytes. Instead, we identify ATF6α as the dominant UPR branch mediating mTOR regulation in this model. Pharmacological inhibition of ATF6α resulted in marked suppression of mTOR activity and reduced Akt phosphorylation, suggesting that ATF6α regulates mTORC1 through an Akt-dependent mechanism.

This finding is consistent with reports from other disease models demonstrating that ATF6α can transcriptionally upregulate components of the mTOR pathway, including Rheb and Akt signaling intermediates [15,16,42]. The cell-level correlation between ATF6α and phosphorylated mTOR observed in this study further supports a functional interaction between ER stress signaling and mTOR activation in Pi*Z hepatocytes.

Importantly, inhibition of mTOR using rapamycin produced multiple beneficial effects in Huh7.5Z cells. mTOR inhibition enhanced autophagic flux, reduced intracellular AAT protein and polymer accumulation, alleviated ER stress, and suppressed apoptotic signaling. These findings are consistent with previous studies showing that autophagy is the primary degradative pathway for mutant Pi*Z AAT aggregates [20,21,43,44] and that pharmacological activation of autophagy improves liver pathology in AATD models [23,24]. Our results further demonstrate that suppression of mTOR signaling reduces activation of UPR pathways and downstream caspase-mediated apoptosis, thereby promoting hepatocyte survival.

**Figure 9:**
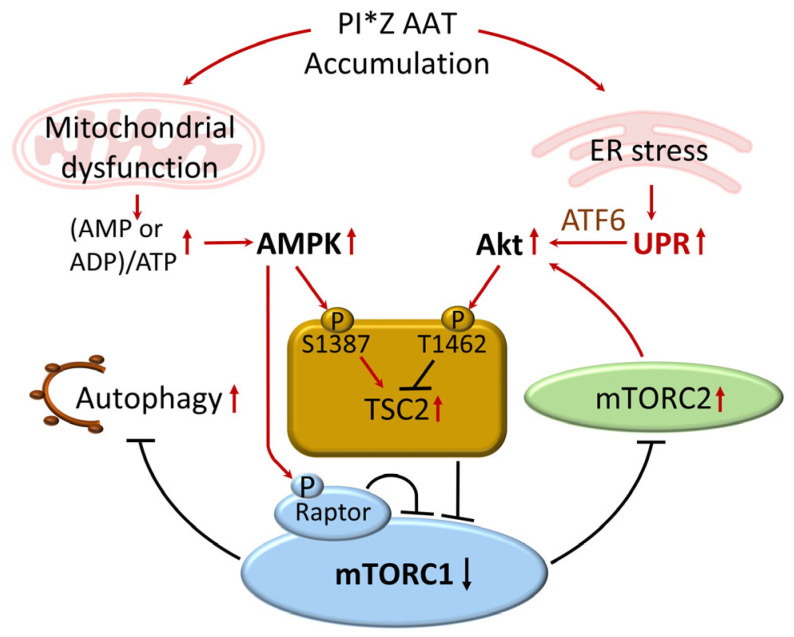
Proposed model of mTOR regulation in Pi*Z hepatocytes. Schematic representation of the integrated signaling network involving mitochondrial dysfunction, AMPK activation, ER stress-induced ATF6α signaling, Akt activation, and adaptive modulation of mTORC1 activity. Arrows indicate activation, and T-shaped lines indicate inhibition. Red and black denote upregulation and downregulation, respectively.

Taken together, our data support a model in which mitochondrial dysfunction activates AMPK to suppress mTORC1, while ER stress through ATF6α and Akt provides counter-regulatory signals that partially restore mTOR activity. This dynamic balance allows hepatocytes to adapt to chronic Pi*Z AAT-induced proteotoxic stress by maintaining sufficient metabolic activity while enhancing autophagy and limiting cell death (Figure 9). The existence of this finely tuned regulatory network suggests that hepatocytes establish a new homeostatic state that prioritizes survival over growth in the setting of persistent ER and mitochondrial stress.

From a therapeutic perspective, our findings reinforce the rationale for targeting mTOR signaling in AATD-associated liver disease. Although mTOR activity is already partially suppressed in Pi*Z hepatocytes, further pharmacological inhibition enhances autophagy and accelerates clearance of toxic AAT aggregates. These results suggest that controlled modulation of mTORC1, particularly in combination with agents that improve mitochondrial function or reduce ER stress, may provide a promising strategy for the treatment of AATD liver disease.

This study has several limitations. First, in this study, many signaling events involve post-translational modifications, which are not always reflected by transcriptional changes; therefore, Western blot analysis was the primary method used to evaluate pathway activation. The quantitative analyses were primarily based on densitometric measurements of Western blot signals, which provide semi-quantitative estimates and may be influenced by experimental conditions. Second, some pharmacological inhibitors used in this study have known off-target effects. For example, dorsomorphin can affect kinases other than AMPK. Although our findings are consistent with AMPK inhibition and supported by siRNA knockdown experiments, future studies using more selective inhibitors or genetic models will be important to further validate these signaling mechanisms. Finally, this study focused on intracellular signaling pathways regulating Z-AAT accumulation and therefore did not directly examine potential effects on AAT secretion into the culture medium, which warrants further investigation.

## 5. Conclusions

In conclusion, this study demonstrates that mTORC1 activity is adaptively suppressed in Pi*Z alpha-1 antitrypsin–deficient hepatocytes and in Pi*Z transgenic mouse liver through coordinated regulation by AMPK, Akt, and ATF6α signaling pathways. Mitochondrial dysfunction activates AMPK to inhibit mTORC1 and promote autophagy, while ER stress–induced ATF6α and Akt signaling partially counterbalance this inhibition. Pharmacological suppression of mTOR further enhances autophagic clearance of intracellular Pi*Z AAT polymers, reduces ER stress, and attenuates apoptosis.

These findings provide new mechanistic insight into how hepatocytes integrate metabolic and proteotoxic stress signals in AATD and identify mTOR regulation as a key adaptive pathway and therapeutic target for AATD-associated liver disease.

## Data Availability

The original contributions presented in this study are included in the article and/or the Supplementary Materials. Further inquiries can be directed to the corresponding author.

## Supplementary Materials

The following supporting information can be downloaded at https://www.mdpi.com/article/10.3390/biom16040506/s1, Supplementary Material S1. Table S1: List of antibodies used for Western blotting and immunofluorescence staining. Table S2. List of qPCR probes. Supplementary Material S2. Original Western Blot Images. Figure S1: Original Western blot images corresponding to Figure 1. Figure S2: Original Western blot images corresponding to Figure 2. Figure S3: Original Western blot images corresponding to Figure 3. Figure S4: Original Western blot images corresponding to Figure 4. Figure S5: Original Western blot images corresponding to Figure 5. Figure S6: Original Western blot images corresponding to Figure 6. Figure S7: Original Western blot images corresponding to Figure 7. Supplementary Material S3. AMPK knockdown by siRNA reduces p-mTOR levels. Figure S8. Knockdown of AMPK by siRNA reduces p-mTOR levels.
