# Supplementary material for: Adaptive Regulation of mTOR Activity by AMPK, Akt, and ATF6 Pathways in Pi*Z Alpha-1 Antitrypsin Deficient Hepatocytes
Source: Biomolecules. 2026 Mar 27;16(4):506. doi: 10.3390/biom16040506 (PMC13114094; doi:10.3390/biom16040506)
Supplement: Supplementary file 1 [file biomolecules-16-00506-s001.zip › biomolecules-4177748-supplementary.pdf]

Adaptive Regulation of mTOR Activity by AMPK, Akt, and ATF6 Pathways in Pi*Z Alpha-1 Antitrypsin Deficient Hepatocytes

Yuanqing Lu, Jungnam Lee, Naweed Mohammad, Mark L. Brantly

Supplementary Material S1

Table S1. List of antibodies used for Western blotting and immunofluorescence staining.

Description	Supplier*	Cat#	Application	dilution
GAPDH Antibody	PTG	10494-1-AP	WB	1:5000
AMPKα (D63G4) Rabbit mAb	CST	5832	WB	1:1000
Phospho-AMPKα (Thr172) (40H9) Rabbit mAb	CST	2535	WB	1:1000
Phospho-Tuberin/TSC2 (Ser1387) Antibody	CST	5584	WB	1:1000
Phospho-Tuberin/TSC2 (Thr1462) (5B12) Rabbit mAb	CST	3617	WB	1:1000
Rheb (E1G1R) Rabbit mAb	CST	13879	WB	1:1000
mTOR (7C10) Rabbit mAb	CST	2983	WB	1:1000
Phospho-mTOR (Ser2448) (D9C2) XP® Rabbit mAb	CST	5536	WB	1:1000
Phospho-mTOR (Ser2448) (D9C2) XP® Rabbit mAb	CST	5536	IF	1:100
Phospho-Raptor (Ser792) Antibody	CST	2083	WB	1:1000
Phospho-S6 Ribosomal Protein (Ser240/244) Antibody	CST	2215	WB	1:1000
Phospho-4E-BP1 (Thr37/46) (236B4) Rabbit mAb	CST	2855	WB	1:1000
LC3B Rabbit Antibody	CST	2775	WB	1:1000
Phospho-Rictor (Thr1135) (D30A3) Rabbit mAb	CST	3806	WB	1:1000
Akt (pan) (C67E7) Rabbit mAb	CST	4691	WB	1:1000
Phospho-Akt (Ser473) (D9E) XP® Rabbit mAb	CST	4060	WB	1:1000
Phospho-Akt (Thr308) (D25E6) XP® Rabbit mAb	CST	13038	WB	1:1000
PERK (C33E10) Rabbit mAb	CST	3192	WB	1:1000
Anti-Phospho-PERK(T982) Rabbit antibody	MS	SAB4301310	WB	1:1000
IRE1α (14C10) Rabbit mAb	CST	3294	WB	1:1000
Anti-Phospho-IRE1alpha(S724) Rabbit antibody	MS	SAB5700519	WB	1:1000
ATF-6 Rabbit antibody	PTG	24169-1-AP	WB	1:2000
ATF6 Mouse Antibody	NB	NBP1-40256	IF	1:100
SQSTM1/p62 (D1Q5S) Rabbit mAb	CST	39749	WB	1:1000
AAT Rabbit Polyclonal Antibody	DAKO	A0012	WB	1:5000
BiP Antibody	CST	3183	WB	1:1000
Caspase-4 Antibody	CST	4450	WB	1:1000
Caspase-9 Antibody	CST	9502	WB	1:1000
Caspase-7 (D2Q3L) Rabbit mAb	CST	12827	WB	1:1000
Human AAT, mAb 2C1	HB	HM2289	IF	1:200
Goat Anti-Rabbit IgG H&L-HRP Conjugate	BR	1706515	WB	1:10000

Goat Anti-Mouse IgG H&L Alexa Fluor 488	AB	ab150113	IF	1:800
Goat anti-Rabbit IgG H&L Alexa fluor 647	AB	ab150079	IF	1:800
Goat anti-Mouse IgG Alexa fluor 647	AB	ab150077	IF	1:800

* CST: Cell Signaling Technology (Danvers, MA, USA);

PTG: Proteintech Group (Rosemont, IL, USA);

MS: MilliporeSigma (Burlington, MA, USA);

NB: Novus Biologicals (Centennial, CO 80112, USA);

DAKO: DAKO (Bath, UK);

HB: Hycult Biotech Inc. (Wayne, PA, USA);

BR: Bio-Rad Laboratories (Hercules, CA, USA);

AB: Abcam (Cambridge, UK).

Table S2. List of qPCR probes.

Gene name	Company	ID	Cat#
18sVIC-TAMRA	Applied Biosystem (Foster City, CA)		4310875
SERPINA	Applied Biosystem (Foster City, CA)	Hs00165475_m1	169784
Atf4	Applied Biosystem (Foster City, CA)	Mm00515324_m1	971994
xbp1t	Applied Biosystem (Foster City, CA)	Mm00457357_m1	1656040
xbp1s	Applied Biosystem (Foster City, CA)	Mm03464496_m1	1251730

Supplementary Material S2. Original Western Blot images

Figure S1. Original Western blot images corresponding to Figure 1.

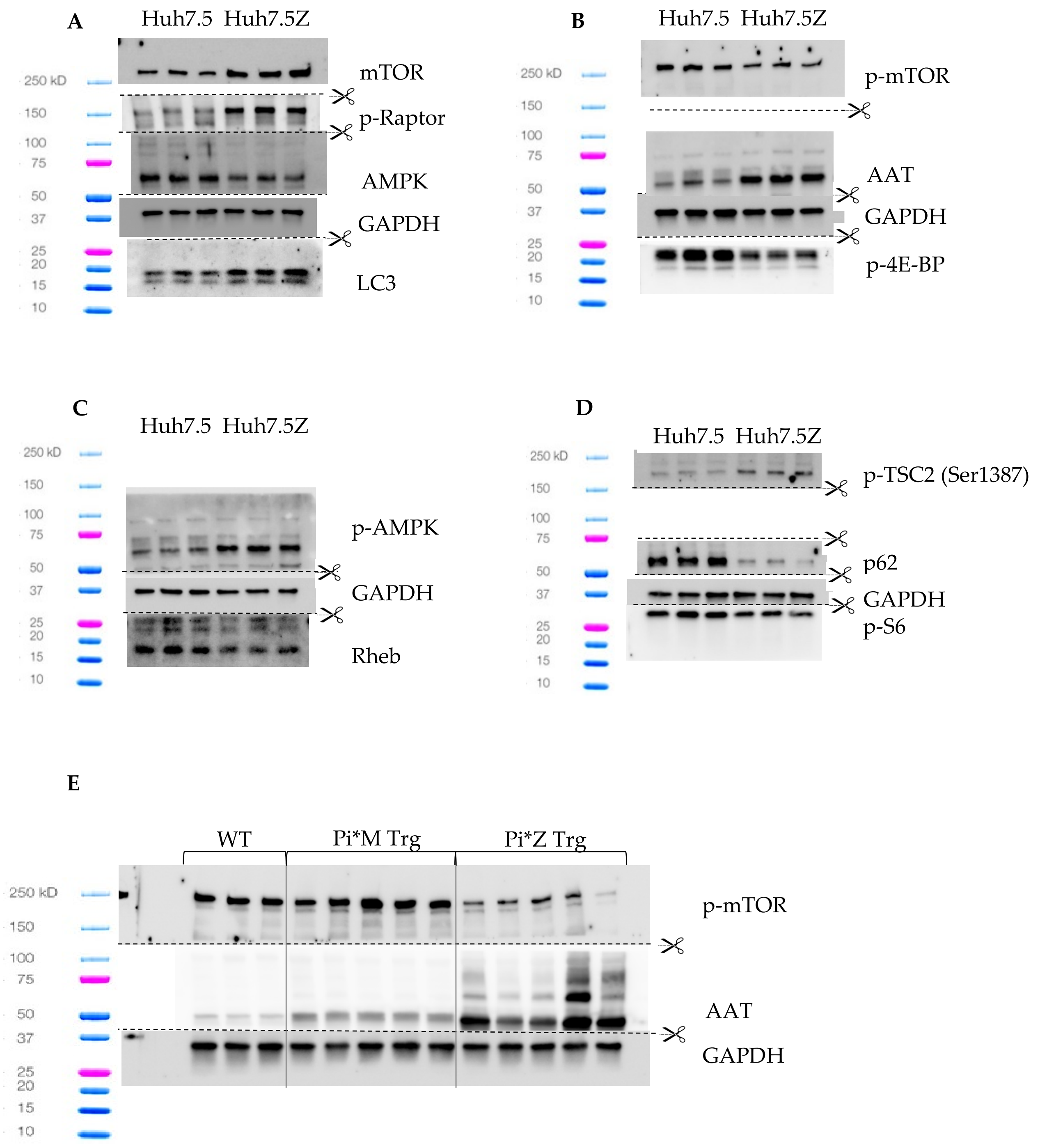

Figure S2. Original Western blot images corresponding to Figure 2

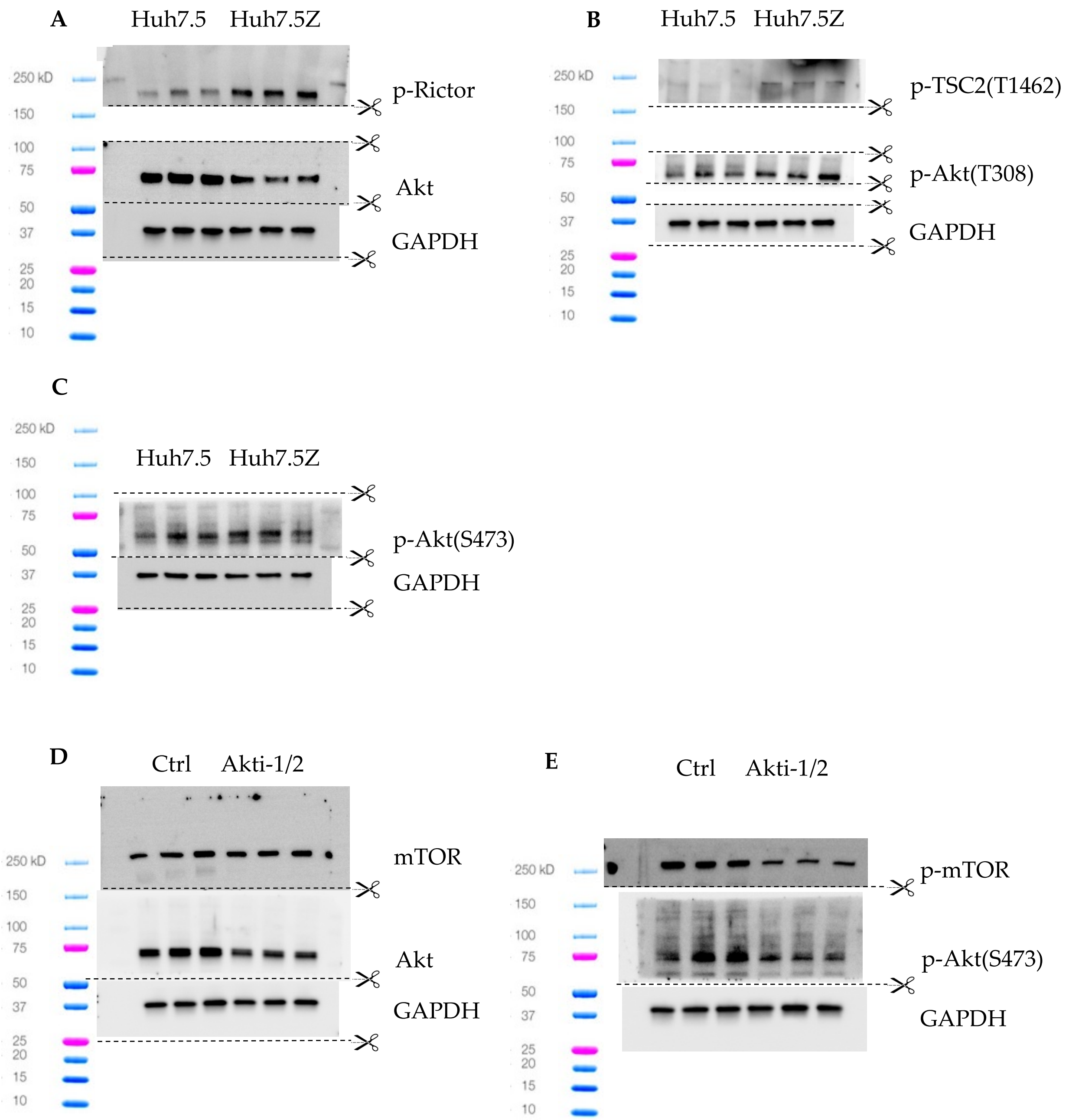

Figure S3. Original Western blot images corresponding to Figure 3

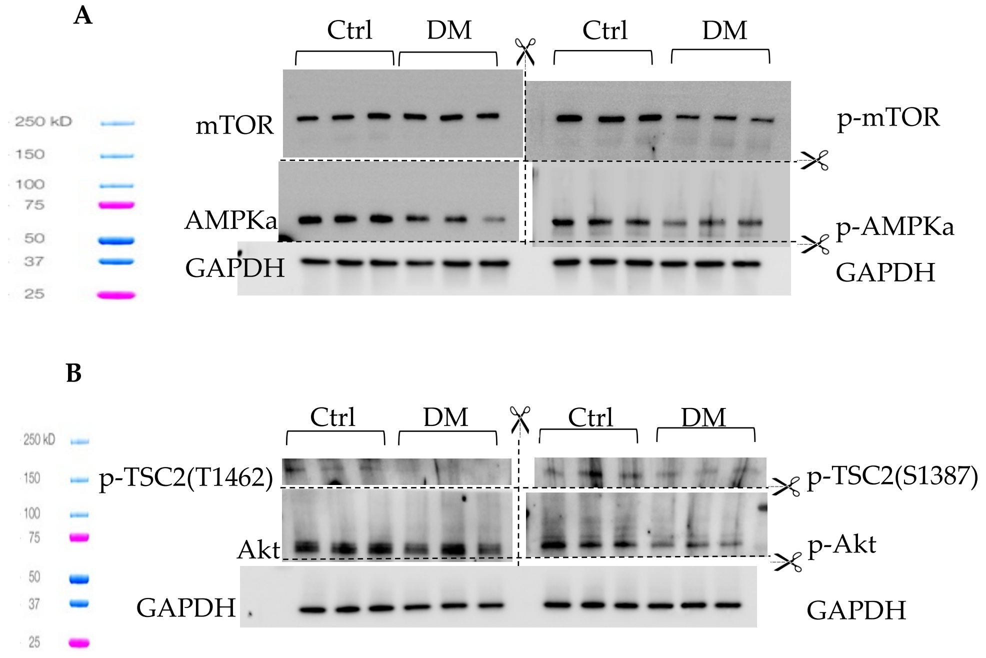

Figure S4. Original Western blot images corresponding to Figure 4

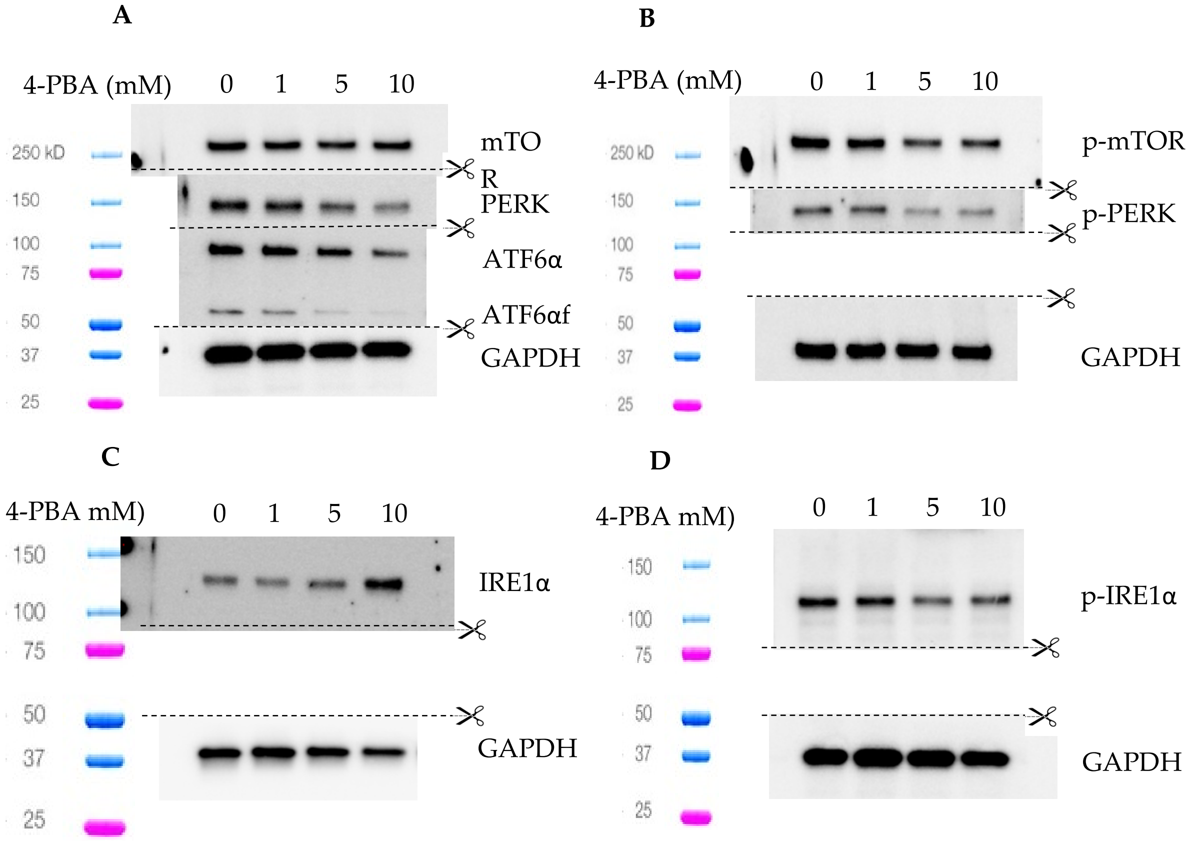

Figure S5. Original Western blot images corresponding to Figure 5

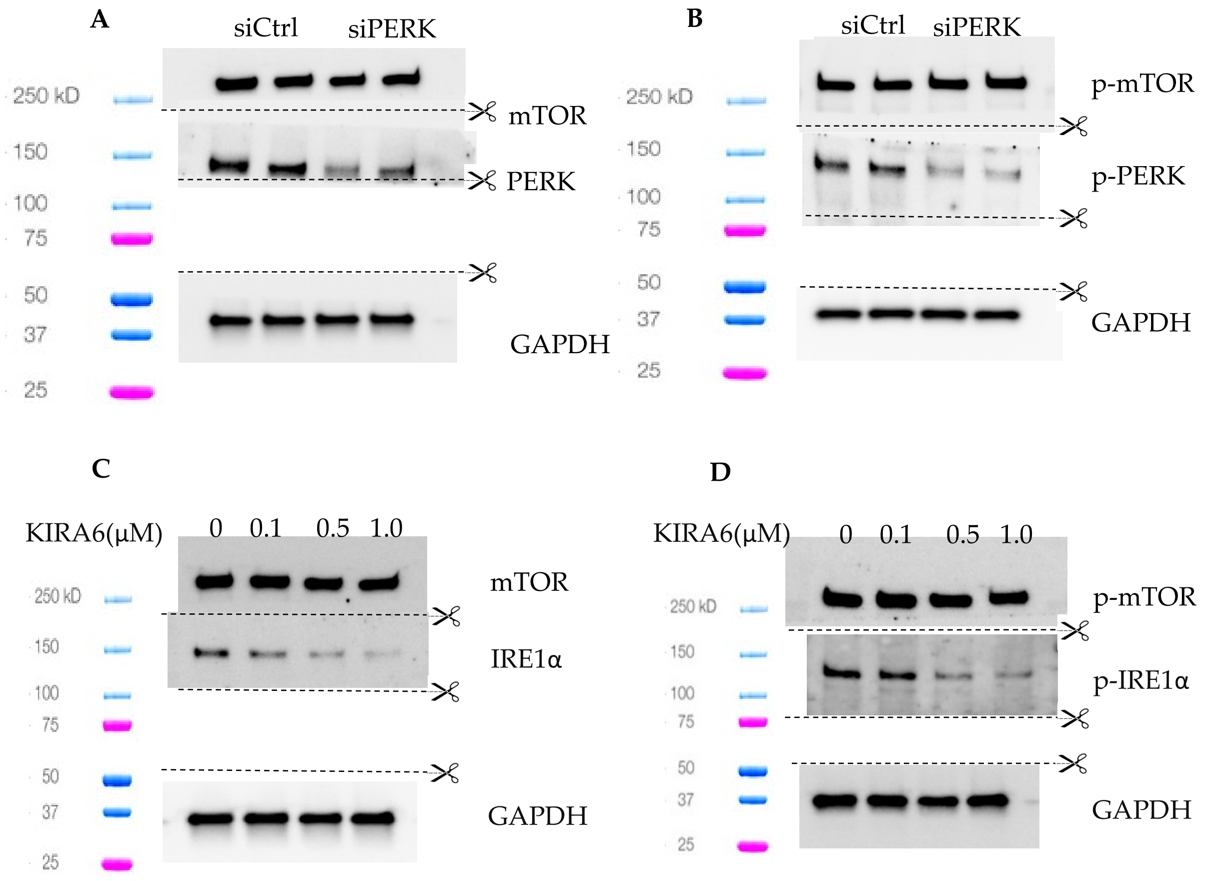

Figure S6. Original Western blot images corresponding to Figure 6

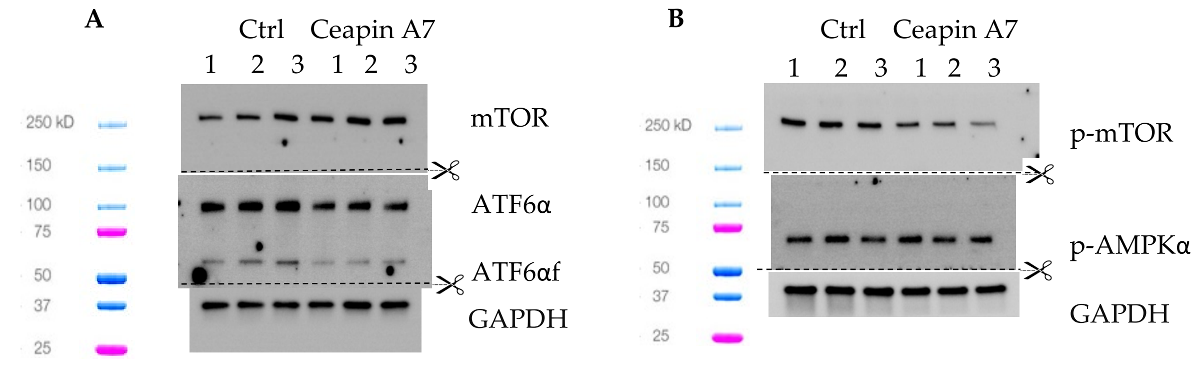

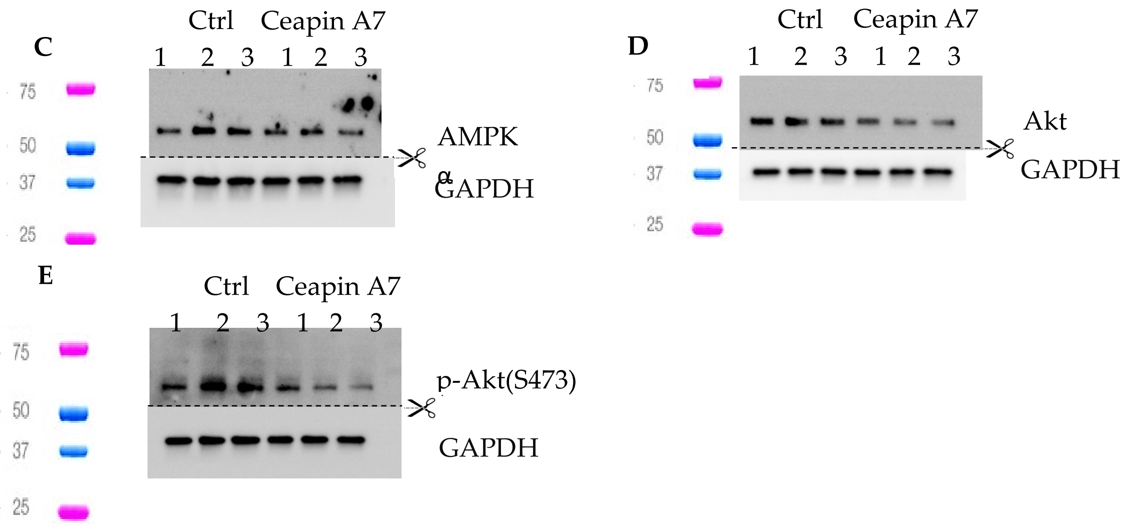

Figure S7. Original Western blot images corresponding to Figure 7

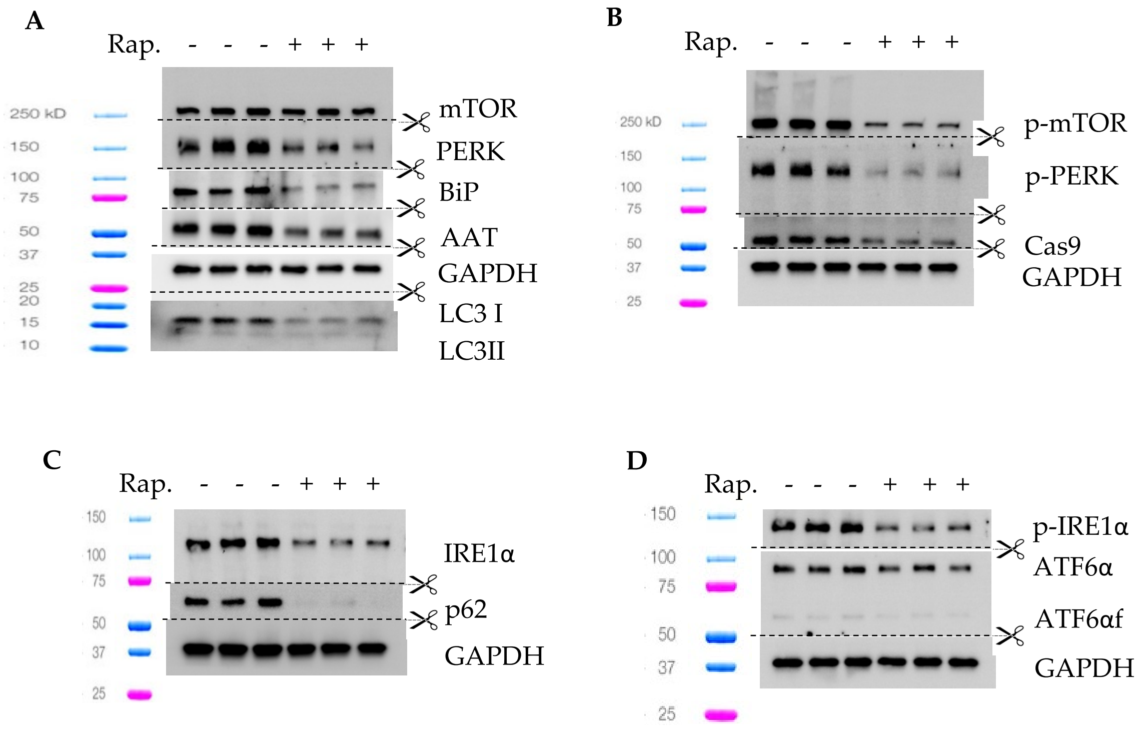

E. Re-probed blots after membrane stripping

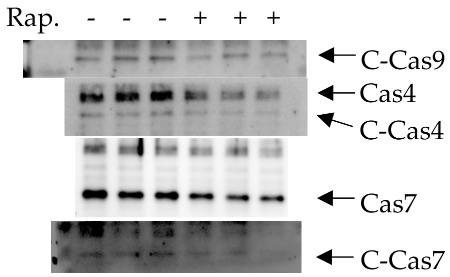

Supplementary Material S3. AMPK knockdown by siRNA reduces p-mTOR levels

Figure S8

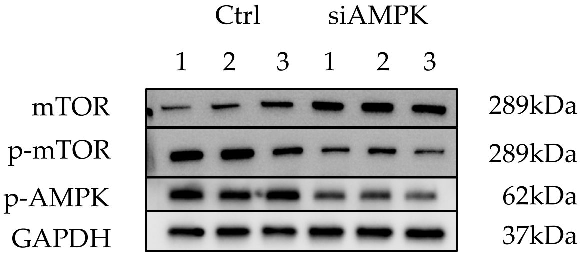

Figure S8. Knockdown of AMPK by siRNA reduces p-mTOR levels.
Huh7.5Z cells were cultured in complete high-glucose DMEM. Prior to transfection, the medium was replaced with antibiotic-free RPMI-1640. Cells were transfected with 50 nM SignalSilence® AMPKα2 siRNA II or control siRNA using SignalSilence® transfection reagent. Five hours after transfection, the medium was replaced with complete DMEM. After 24 h, cell lysates were collected and subjected to Western blot analysis. The results showed that AMPK siRNA reduced p-AMPK levels. Notably, p-mTOR levels were also reduced. Significant cell death was observed in siAMPK-treated Huh7.5Z cells but not in the control group.
